# A de novo mutation of ADAMTS8 in a patient with Wiedemann–Steiner syndrome

**DOI:** 10.1186/s13039-023-00654-0

**Published:** 2023-08-30

**Authors:** Sifeng Wang, Shuyuan Yan, Jingjun Xiao, Ying Chen, Anji Chen, Aimin Deng, Tuanmei Wang, Jun He, Xiangwen Peng

**Affiliations:** Hunan Provincial Key Laboratory of Regional Hereditary Birth Defects Prevention and Control, Changsha Hospital for Maternal and Child Health Care Affiliated to Hunan Normal University, Changsha, China

**Keywords:** Wiedemann–Steiner syndrome, *KMT2A*, *ADAMTS8*, Early teething, Rapid tooth replacement, Dysplastic enamel

## Abstract

**Background:**

Wiedemann–Steiner syndrome (WDSTS) is a rare autosomal dominant disorder caused by mutations in the *KMT2A* gene and is usually characterized by hairy elbows, short stature, developmental delay, intellectual disability and obvious facial dysmorphism.

**Case presentation:**

Here, we report a 5-year-old girl with clinical features similar to WDSTS, including postnatal growth delay, retarded intellectual development, and ocular hypertelorism. Through whole-exome sequencing (WES), a frameshift variant of *KMT2A* was found in the patient but not in her parents’ genomic DNA. By bioinformatics analysis, the *KMT2A* variant was demonstrated to be the top candidate pathogenic variant for the clinical phenotype consistent with WDSTS. Moreover, a duplication of exon 1 in *ADAMTS8* (belonging to the zinc metalloproteinase family) was found in the genomic DNA of this patient, which may be responsible for the characteristics that are different from those of WDSTS, including early teething, rapid tooth replacement, and dysplastic enamel.

**Conclusions:**

From the above results, we propose that in our patient, the frameshift variant in *KMT2A* is the main reason for the WDSTS phenotype, and the unreported mutation in *ADAMTS8* may be the candidate reason for other characteristics that are different from those of WDSTS. Therefore, this study not only provides a new *KMT2A* variant associated with WDSTS but is also a reminder that combined mutations may be present in a case with more characteristics than those seen in WDSTS.

**Supplementary Information:**

The online version contains supplementary material available at 10.1186/s13039-023-00654-0.

## Introduction

As a rare autosomal dominant genetic disease, Wiedemann–Steiner syndrome (WDSTS, OMIM 605130) was first described by Wiedemann et al. in 1989 and subsequently was reported in approximately 20 papers [1–21]. Patients with WDSTS presented with short stature; general developmental delay; inability to grow; intellectual disability; language development delay; aggressive behaviour; seizures; hypotonia; tapered fingers; deformed flexion of the little finger; short toes; wide base gait; delayed bone maturation; and facial dysmorphisms, such as hypertelorism, high palate, thin upper lip, inner canthal epidermis, long middle and low ears, broad nasal bridge, depressed nasal tip, wide nose, strabismus, down slanting palpebral fissures and long eyelashes [1, 10, 22]. Here, we report a patient with a *KMT2A* frameshift variant with intellectual disability and facial abnormalities similar to those of previously reported patients with *KMT2A* mutations [1, 4, 23, 24]. In addition, distinct phenotypes different from WDSTS, such as early teething, rapid tooth replacement, dysplastic enamel and the duplication of exon 1 in the zinc metalloproteinase *ADAMTS8,* were observed in the patient but not in her parents. Our research has enriched the knowledge of WDSTS with *KMT2A* mutations and reported a de novo mutation in *ADAMTS8,* which may be the candidate reason for the distinct phenotypes different from WDSTS observed in our patient.

## Results

A 5-year-old girl (height 94.4 cm, weight 12.5 kg, both 3 percentile) was admitted to Changsha Hospital for Maternal & Child Health Care because of developmental retardation and low muscle tension. Her conception, delivery, birth height (50 cm) and birth weight (3.2 kg) were normal. Upon physical examination, the clinical features of the patient were described as hypertrichosis cubiti, postnatal growth retardation, delayed intellectual development, short and broad fingers, hypotonia of the lower limbs and distinctive facial appearances such as ocular hypertelorism. From these characteristics, the patient was first diagnosed with WDSTS, although she had no family history of this disease.

**Fig. 1:**
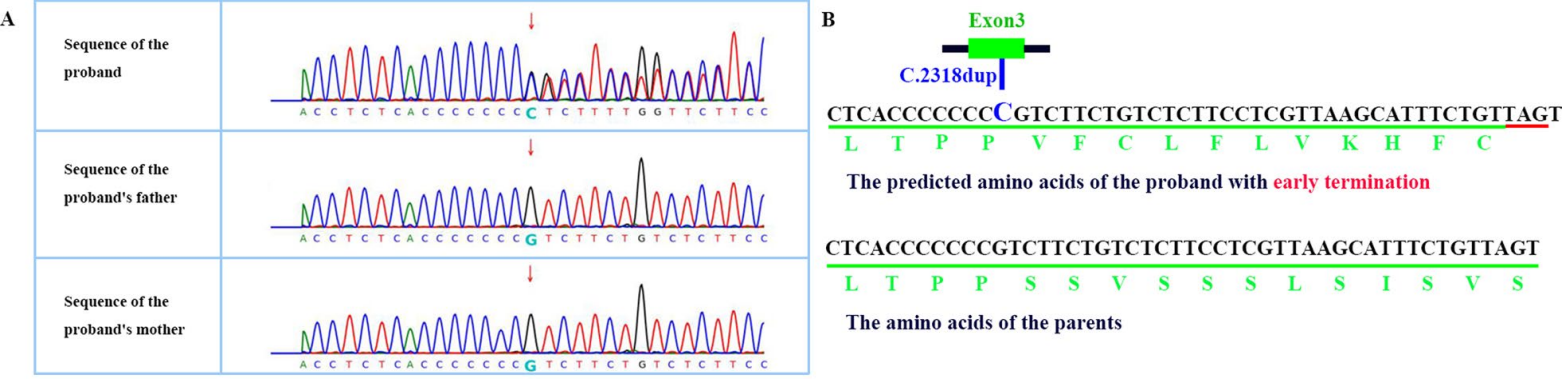
**A** Frameshift variant in the proband but not in her parents confirmed by first generation sequencing; **B** The predicted change of amino acids of the proband

To further confirm this diagnosis at the molecular level, whole-exome sequencing (WES) and whole-genome copy number variation (CNV-seq) detection were performed on the genomic DNA of the patient and her parents. Data were analysed by Berry Gene’s Verita Trekker® variant site detection system and Enliven® Variant Annotation Interpretation System. One frameshift variant of the gene *KMT2A* was detected in the subject. The c.2318:p.S774Vfs*12 variant of the gene *KMT2A* is believed to be a pathogenic mutation site (Fig. 1A, B), which causes changes in the open reading frame of the gene and leads to malformation of the KMT2A protein associated with the autosomal dominant genetic disease WDSTS. After bioinformatics filtering, we obtained another 97 candidate variations (Additional file 1: Table S1) in genes such as *GLI2* and *RAD50*, but the phenotypes of patients with mutations in these genes did not match our case. Therefore, we predicted that the observed phenotypes were caused by the c.2318:p.S774Vfs*12 variant in *KMT2A* in our patient with intellectual disability, intellectual developmental delay, wide eye distance, and hypotonia.

**Fig. 2:**
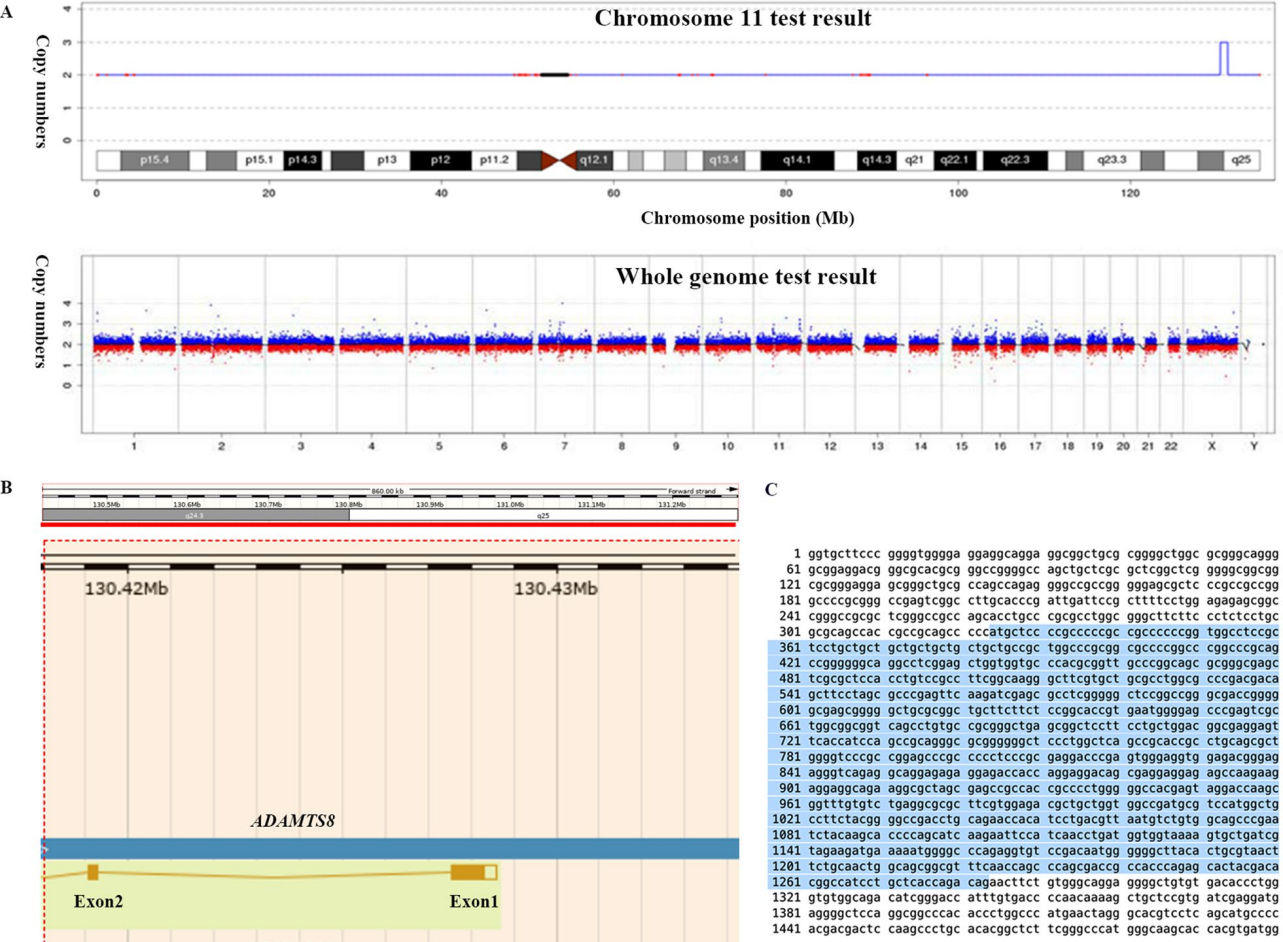
**A** Genome copy number variants (CNVs) with 0.86 Mb repeat:seq [hg19]dup(11(q24.3q25)chr11:g.130420000_131280000dup11chromosome q24.3-q25. **B**. The sequencing map of repeat sequence in exon 1 of *ADAMTS8*. **C** The repeat sequence in exon 1 of *ADAMTS8* labelled with blue

Abnormal teeth in our patient were also described, including early teething, rapid tooth replacement, and dysplasia of dentition not reported in other cases with *KMT2A* mutations. To find the candidate reason for these atypical characteristics, we further analysed the sequencing data, and a de novo duplication of exon 1 in *ADAMTS8* which was found in the patient but not in her parents. Repetitive sequences were detected in exon 1 of *ADAMTS8* (Fig. 2), which may cause malformation and dysfunction of the ADAMTS8 protein. No case of a mutation in *ADAMTS8* has been reported to date. We cannot exclude the possibility that this mutation may be the candidate reason for the atypical phenotype in our patient.

## Discussion

WDSTS is a rare syndrome with the main clinical phenotypes of postnatal growth retardation, intellectual disability, hypertrichosis cubiti, and dysmorphic facial features. However, similar phenotypes have also been described in other diseases, such as Kabuki, Rubenstein-Taybi, and Floating-Harbor syndromes [25–27]. The overlapping of these syndromes with WDSTS presents a challenge for clinical diagnosis. With the development of genome-sequencing technology, this problem has been partially resolved, and an increasing number of genetic diseases have been diagnosed. To date, 644 unique *KMT2A* variant annotations have been identified in 274 publications (Bibliome). There are currently more than 20 papers reporting new mutations in the *KMT2A* gene. The ClinVar database has reported nearly 100 mutations and small indels [3, 20], of which 43 are small frameshift indels, 39 are missense mutations and 4 are splicing mutations. Moreover, nearly 60% of mutations are located in exons 3 and 27 [5]. The mutation in our case is located within exon 3 of the *KMT2A* gene. By using molecular diagnosis, the de novo mutation in our patient was confirmed to be unique, although similar mutations in different exons have been reported in other Chinese children [15].

Most patients with *KMT2A* mutations share similar clinical features, while other accompanying phenotypes have also been reported in patients with WDSTS. Congenital immunodeficiency and microcephaly were observed in a boy with WDSTS [12]. The additional feature of broad toes was also described in a boy who was the first reported patient with WDSTS from India [13]. Right preaxial polydactyly was first found in a 12-year-old Japanese boy with WDSTS, but the relationship between his *KMT2A* mutation and this new phenotype was not confirmed [17]. Distinct phenotypes, including early teething, fast tooth replacement, and dysplastic enamel, not found in WDSTS were also observed in our patient with a frameshift variant in *KMT2A*. Therefore, it is unclear whether these unreported phenotypes were caused by the *KMT2A* mutation. We further analysed the sequencing data and found repetitive sequences in exon 1 of *ADAMTS8* in our patient. This de novo mutation may be the candidate reason for these new phenotypes that are different from the main features of WDSTS. To date, no case of mutation in *ADAMTS8* has been reported, and our proposal cannot be confirmed by previous reports. However, we hypothesize that mutations in other genes cause additional phenotypes in patients with *KMT2A* mutations and WDSTS, which should be considered in the diagnosis of patients with more phenotypes than typical in WDSTS in the future.

## Methods

Genomic DNA was extracted from the peripheral blood of the patient and her parents. We performed whole-genome sequencing on the NovaSeq 6000 platform (Illumina, San Diego, USA) with a depth of 20X. The coverage of the proband, father and mother were 98.95%, 99.01%, and 98.87%, respectively. Raw image files were processed using CASAVA v1.82 for base calling and generating raw data. The sequencing reads were aligned to the human reference genome (hg38/GRCh38) using the Burrows–Wheeler Aligner tool, and PCR duplicates were removed by using Picard v1.57 (http://picard.sourceforge.net/). The Verita Trekker® Variants Detection System by Berry Genomics and GATK software (https://software.broadinstitute.org/gatk/) were employed for variant calling. Variant annotation and interpretation were conducted by ANNOVAR (Wang, et al. 2010) and the Enliven® Variant Annotation Interpretation System authorized by Berry Genomics. Annotation databases mainly include the following:

i) Human population databases, such as gnomAD (http://gnomad.broadinstitute.org/), the 1000 Genomes Project (http://browser.1000genomes.org), the Berry big data population database, and dbSNP (http://www.ncbi.nlm.nih.gov/snp).
ii) In silico prediction algorithms, such as SIFT (http://sift.jcvi.org), FATHMM (http://fathmm.biocompute.org.uk), Mutation Assessor (http://mutationassessor.org), CADD (http://cadd.gs.washington.edu), and SPIDEX (Xiong et al., Science 2015).
iii) Disease and phenotype databases, such as OMIM (http://www.omim.org), ClinVar (http://www.ncbi.nlm.nih.gov/clinvar), HGMD (http://www.hgmd.org), and HPO (https://hpo.jax.org/app/).

The variants were classified into five categories – “pathogenic”, “likely pathogenic”, “uncertain significance”, “likely benign” and “benign” – according to the American College of Medical Genetics and Genomics (ACMG) guidelines for interpretation of genetic variants (Richards et al., 2015). Variants with minor allele frequencies (MAFs) < 1% in the exonic region or with splicing impact were selected for further interpretation, considering the ACMG category, evidence of pathogenicity, and clinical synopsis and inheritance model of the associated disease.

For trio analysis, potential monogenetic inheritance patterns, including de novo, autosomal recessive, autosomal dominant, X-linked recessive inheritance and imprinted gene variation, were analysed. Full penetrance was assumed for the potentially causal variants, and variants that were found in the parents or were recorded in any of the abovementioned databases or in our in-house control exomes were excluded as the aetiology. Once a variant was considered to be the aetiology of a recessive disorder, manual inspection for coverage and additional variants of the entire coding domain was undertaken using Integrated Genomics Viewer.

### Supplementary Information

**Additional file 1: Table S1. **Candidate gene variations in our case.

## Data Availability

The original contributions presented in the study are included in the article and further inquiries of raw data can be directed to the corresponding author.
